# Excessive Labeling Technique Provides a Highly Sensitive Fluorescent Probe for Real-time Monitoring of Biodegradation of Biopolymer Pharmaceuticals in vivo

**Published:** 2014

**Authors:** S. S. Terekhov, I. V. Smirnov, O. G. Shamborant, M. A. Zenkova, E. L. Chernolovskaya, D. V. Gladkikh, A. N. Murashev, I. A. Dyachenko, V. D. Knorre, A. A. Belogurov, N. A. Ponomarenko, S. M. Deyev, V. V. Vlasov, A. G. Gabibov

**Affiliations:** Shemyakin-Ovchinnikov Institute of Bioorganic Chemistry, Russian Academy of Sciences, Miklukho-Maklaya Str., 16/10, GSP-7, Moscow, Russia, 117997; Institute of Chemical Biology and Fundamental Medicine, Siberian Branch of the Russian Academy of Sciences, Lavrentiev Ave., 8, 630090, Novosibirsk, Russia; Branch of the Shemyakin-Ovchinnikov Institute of Bioorganic Chemistry, Russian Academy of Sciences, Pushchino, 142290, Moscow region, Russia; Kazan Federal University, Kremlevskaya str. 18, 420008, Kazan, Republic of Tatarstan, Russia; Institute of Gene Biology, Russian Academy of Sciences, Vavilova Str., 34/5, 119334, Moscow, Russia

**Keywords:** fluorescent probe, biodegradation, pharmacokinetics, in vivo bioimaging, self-quenching, butyrylcholinesterase, proteolysis

## Abstract

Recombinant proteins represent a large sector of the biopharma market. Determination of the main elimination pathways raises the opportunities to significantly increase their half-lives *in vivo*. However, evaluation of biodegradation of pharmaceutical biopolymers performed in the course of pre-clinical studies is frequently complicated. Noninvasive pharmacokinetic and biodistribution studies in living organism are possible using proteins conjugated with near-infrared dyes. In the present study we designed a highly efficient probe based on fluorescent dye self-quenching for monitoring of *in vivo *biodegradation of recombinant human butyrylcholinesterase. The maximum enhancement of integral fluorescence in response to degradation of an intravenously administered enzyme was observed 6 h after injection. Importantly, excessive butyrylcholinesterase labeling with fluorescent dye results in significant changes in the pharmacokinetic properties of the obtained conjugate. This fact must be taken into consideration during future pharmacokinetic studies using *in vivo *bioimaging.

## INTRODUCTION

Modern pharmacokinetics is a technology-intensive field that utilizes cutting-edge noninvasive approaches, such as single-photon emission computed tomography (SPECT), positron emission tomography (PET), and *in vivo *bioimaging not only for determining pharmacokinetic parameters but also for studying biodistribution and evaluating the accumulation profiles of pharmaceuticals [1]. Since recombinant protein-based pharmaceuticals are widely used in treatment of serious diseases such as cancer [2, 3], autoimmune diseases [4], and blood disorders [5], it is extremely important to examine their biodistribution and biodegradation during preclinical studies. The new infrared fluorescent dyes with a high brightness allow working in the tissue transparency window (700–900 nm), are commercially available, safe, and efficient, which makes fluorescence bioimaging one of the most widely used imaging techniques [6, 7]. The imaging data can reveal pharmaceutical accumulation compartment [8] and pharmacokinetic parameters of elimination [9]. The relationship between accumulation and biodegradation is especially crucial for the recombinant protein-based pharmaceuticals with specific activity. The knowledge about pharmaceutical biodegradation is also of great interest as it allows one to determine the main elimination routes.

The aim of this study was to design a probe that enables determination of the main biodegradation compartments of a recombinant protein. Recombinant human butyrylcholinesterase, a bioscavenger of organophosphorus agents, was used as an example of the protein-based biological antidote against nerve-agent poisoning [10, 11]. The approach employed is based on self-quenching of fluorophores [12]. The essence of this phenomenon is that fluorescence quenching is typical of fluorophore molecules characterized by a small Stokes’ shift (~20–30 nm) and situated at a distance of less than 10 nm from one another. Quenching effectiveness depends, among other factors, on the aggregation tendency of fluorophore molecules due to their π-π and hydrophobic interactions [13]. Thus, excessive modification of a protein-based pharmaceutical with an infrared fluorophore having the self-quenching effect gives rise to a conjugate with “switched off” fluorescence, while its degradation and the formation of peptide products enhances the integral fluorescence. This approach is currently used for tumor imaging based on the presence of MMP activity or presence of specific or hyperexpressed receptors [14-18] and to detect the activity of proteolytic antibodies against HIV surface protein, gp120 [19]. In this study, we proposed excessive labeling of recombinant butyrylcholinesterase to visualize the compartments responsible for biodegradation and elimination of the pharmaceutical *in vivo *and to evaluate the parameters of enzyme biodistribution and biodegradation.

## MATERIALS AND METHODS

**Protein-based pharmaceuticals**

Tetrameric recombinant human butyrylcholinesterase (rhBChE) was produced in CHO-K1 cells transfected with a pFUSE PRAD-F2A-BChE construct, which results in simultaneous expression of the PRAD tetramerization peptide gene and the human butyrylchoninesterase gene. rhBChE was purified by affinity chromatography on a procainamide-Sepharose XK10/50 column (GE Healthcare, USA) followed by ion exchange chromatography on a MonoQ 5/50 column (GE Healthcare, USA). Polyacrylamide gel electrophoresis followed by Coomassie staining and Karnovsky and Roots’s staining to detect specific butyrylcholinesterase activity [20] showed 95% protein purity. Commercially available KLH and BSA proteins were purchased from Sigma Aldrich.

**Synthesis of pharmaceuticals based on fluorescently labeled proteins**

The proteins were labeled with various NHS-activated fluorophores of the cyanine family: Sulfo-Cyanine5 (sCy5) and Sulfo-Cyanine7 (sCy7) (Lumiprobe). The conjugation procedure was performed in 0.1 M NaHCO_3_ in accordance with the manufacturer’s protocol. Reaction products were removed from the fluorescently labeled proteins by gel filtration chromatography on a HiTrap Desalting column (GE Healthcare, USA). The fluorescence of the pharmaceuticals was measured on a Varioscan Flash instrument (Thermo Scientific). To determine the maximum relative fluorescence enhancement, the protein-based pharmaceuticals were subjected to proteolysis with a solution containing a protease mixture (1 mg/ml proteinase K (Fermentas) and 2 mg/ml subtilisin Carlsberg) in phosphate buffered saline pH 7.4 at 37°C for 4 h. The completeness of proteolysis was monitored fluorimetrically according to saturation attainment on the fluorescence intensity (RFU) vs. time curve. The relative fluorescence enhancement (F_max_) was calculated as the ratio between the difference of fluorescence intensity after proteolytic digestion (Fenz) and fluorescence intensity before proteolytic digestion (F_0_): F_max_ = (F_enz_ - F_0_)/F_0_*100%. The modification degree of the samples (N), i.e., the number of fluorophore groups per protein molecule, was determined by the absorbance measurements of the solutions at 280 nm (E^1%^=18) and 760 nm; the molar extinction coefficient of sCy7 was assumed to be 240600 M^-1^cm^-1^.

**Determination of the pharmacokinetic parameters of rhBChE-sCy7 conjugates**

rhBChE samples (without a fluorescent label, low-labeled rhBChE-sCy7 ON and excessively conjugated rhBChE-sCy7 OFF) were intravenously injected at a dose of 200 μg/mouse into three groups of BALB/c mice with 6 animals per group to assess blood concentrations of rhBChE conjugates. rhBChE concentration in mouse blood serum was determined according to its activity using the Ellman’s method [21]. The pharmacokinetic characteristics of rhBChE samples were obtained from fitting the serum rhBChE vs time curve using the two-compartment model [10].

**In vivo imaging experiments**

The biodistribution and the degradation pattern of rhBChE were determined using rhBChE-sCy7 OFF and rhBChE-sCy7 ON. The rhBChE-sulfo-Cyanine7 conjugates were intravenously injected into BALB/c mice at a dose of 500 μg/mouse. An In Vivo MS FX PRO small animal imaging system (Bruker) was used to visualize the distribution of BChE and its degradation products. The excitation (730 nm) and emission (790 nm) filters were used to detect the fluorescence of sCy7.

## RESULTS AND DISCUSSION

The application of excessively labeled protein fluorescence quenching with subsequent fluorescence enhancement via proteolytic degradation has been successfully demonstrated in studies with a low level of proteolytic activity [22] or by analyzing low enzyme levels [23]. It is evident that fluorescein cannot be used as a source of analytical signal in *in vivo *studies, since organs and tissues provide a high background signal. To eliminate this disadvantage, we conjugated protein molecules to sCy5 and sCy7 dyes under various conditions. We also suggested that the use of red and near-infrared fluorophores results in more sensitive probes for proteolytic activity as fluorescence enhancement would be more efficient. We used the conventional substrate proteins utilized to analyze nonspecific proteolytic activity (bovine serum albumin (BSA)) and hemocyanin (KLH)) and butyrylcholinesterase—a pharmaceutical utilized as a biological antidote against nerve-agent poisoning. As a result, KLH-sCy5, BSAsCy5, rhBChE-sCy5, BSA-sCy7, rhBChE-sCy7 ON and rhBChE-sCy7 OFF specimens were obtained (Table 1).

**Table 1:** Properties of the fluorescent dye labeled proteins used in the present study

Conjugate	*N*	F_0_, RFU	F_enz_, RFU	F_max_
BSA-FITC_(Voss et al.)_	25	-	-	3450
KLH-sCy5	380-750	6.25	1140	18100
BSA-sCy5	6.7	2.37	1680	70800
rhBChE-sCy5	30	6.17	1750	28300
BSA-sCy7	6.5	1.8	660	36500
rhBChE-sCy7 OFF	32	2.71	597	21900
rhBChE-sCy7 ON	1	50	50.05	0.1

N – modification degree; F_0_ – fluorescence before proteolytic digestion; F_enz_ – fluorescence after proteolytic digestion.

The conjugates were subjected to proteolysis to determine the fluorescence enhancement efficiency. All the fluorescent substrates exhibited high levels of maximum enhancement of fluorescence (F_max_) (Table 1), which exceeded those of the conventional BSA–FITC substrate. The rhBChE-sCy5 specimen showed the highest efficiency (fluorescence increased over 700-fold).

**Fig. 1:**
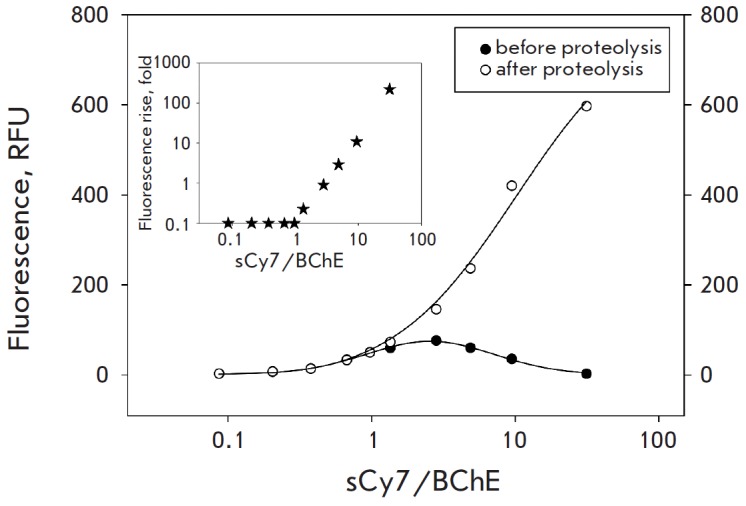
Analysis of rhBChE-sCy7 conjugates with different modification degrees

The fluorescence of the rhBChE-sCy7 ON specimen remained unchanged before and after proteolytic digestion, while the fluorescence of the rhBChE-sCy7 OFF specimen was significantly quenched and increased 220-fold after proteolytic digestion. Thus, the rhBChE-sCy7 OFF specimen can act as a probe to assess the proteolytic degradation of rhBChE in pharmacokinetic experiments. We analyzed several variants of excessively labeled butyrylcholinesterase to design a fluorescent probe with maximum efficiency. We relied on two selection criteria: relative fluorescence intensity enhancement and specific activity of the modified enzyme (*Fig. 1*). The variant with a modification degree N=32 was eventually chosen, since more than 70% of its specific activity was retained.

**Pharmacokinetic studies of rhBChE-sCy7 ON and rhBChE-sCy7 OFF specimens**

**Fig. 2:**
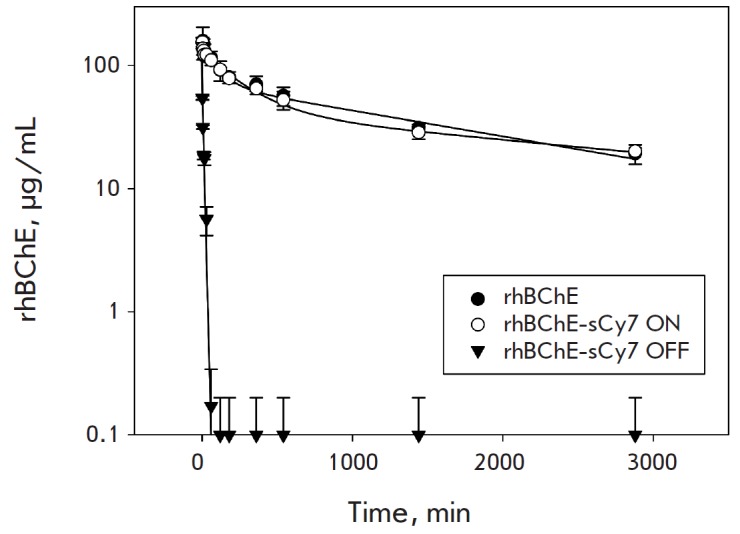
Analysis of the pharmacokinetic parameters of elimination of rhBChE and rhBChE-sCy7 conjugates from serum

Studying the pharmacokinetic parameters of the rhBChE-sCy7 ON and rhBChE-sCy7 OFF specimens, we used a unique opportunity to assess the butyrylcholinesterase level in the blood flow simultaneously using the kinetic and fluorescent methods. Indeed, the pharmacokinetic parameters of protein-based pharmaceuticals are usually assessed using either a direct radioactive method or indirect methods (e.g., ELISA). However, all these methods often indicate only that there is a protein fragment (containing a radioactive label or specific antibody–epitope binding, respectively) but do not prove the presence of an active protein-based pharmaceutical. In the case of butyrylcholinesterase, only the active enzyme acts as a biological antidote; hence, the actual pharmacokinetic parameters of the pharmaceutical can be evaluated by observing the changes in its activity in the blood. The fluorescent probe shows the existence of the integral protein as well as its fragments. Hence, comparison of the elimination profiles observed using two different methods demonstrates pharmaceutical degradation against a background of elimination from the organism.

**Table 2:** Pharmacokinetic parameters of rhBChE and rhBChE-sCy7 conjugates

Parameter	rhBChE	rhBChE-sCy7 ON	rhBChE-sCy7 OFF
τ_1/2_ disr, min	100±40	140±50	6±2
τ_1/2_ el, min	1600±300	2200±400	
MRT, min	2400±600	2700±700	9±3

Fig. 2 and Table 2 show the identity of the elimination pattern and parameters of rhBChE-sCy7 ON to the respective unmodified enzyme, while the rhBChEsCy7 OFF specimen is characterized by drastic changes in its behavior in the organism. The elimination rate greatly increases (Table 2).

**Fig. 3:**
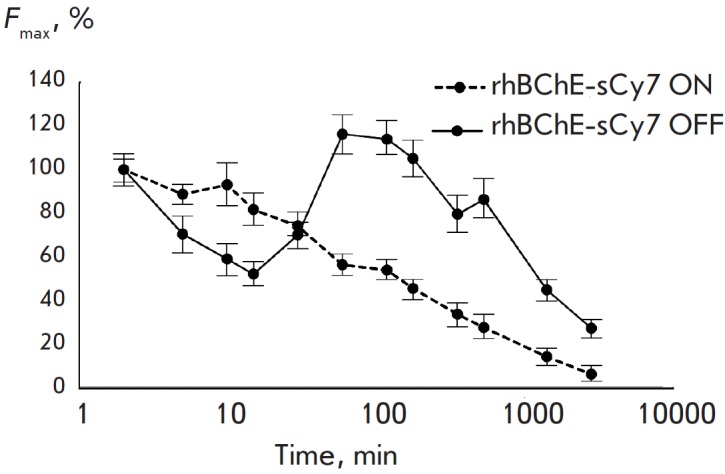
Analysis of the elimination kinetics of rhBChE-sCy7 conjugates observed by sCy7 fluorescence

**Fig. 4:**
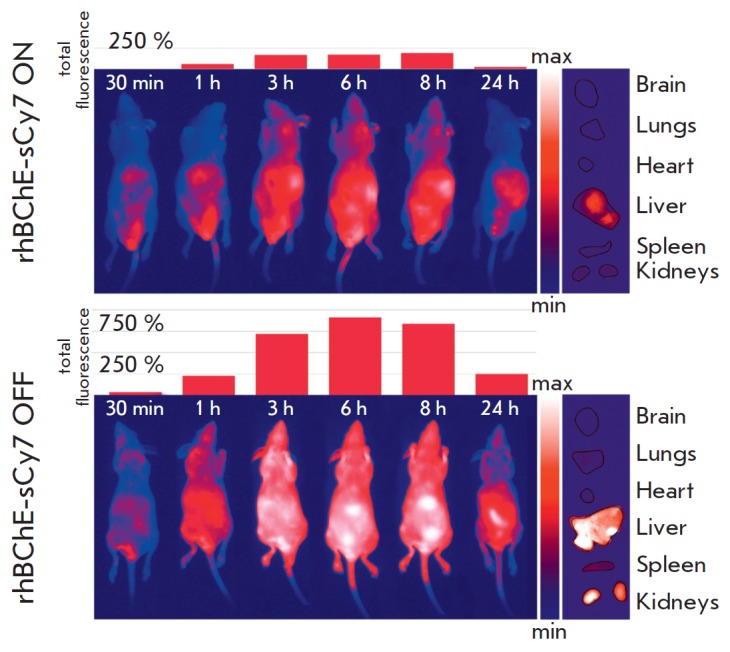
Biodistribution analysis of rhBChE-sCy7 conjugates using fluorescent bioimaging

It was found by analyzing the elimination profiles observed by fluorescence in different time points after the pharmaceutical had been administered (*Fig. 3*) that the maximum fluorescence level is observed 1.5–8 h after administration. It is fair to say that within this period pharmaceutical accumulation reaches the highest level in liver where the enzyme is actively degraded, which results in an increase in fluorescence (*Fig. 4*). The pharmacokinetics of elimination of rhBChE-sCy7 ON with fluorescent detection (*Fig. 3*) does not significantly differ from a similar elimination curve for rhBChEsCy7 ON detected according to the enzymatic activity (*Fig. 2*). Hence, fluorescent detection of enzyme distribution adequately shows the accumulation of rhBChE in a certain compartment. Meanwhile, the comparison of these curves for the rhBChE-sCy7 OFF specimen clearly indicates that two different processes take place: 1 – rapid elimination of rhBChE-sCy7 OFF from the blood flow (which is unrelated to the degradation of rhBChE-sCy7 OFF and results in a rapid decrease in rhBChE activity in blood but is not accompanied by fluorescence intensity enhancement); 2 – slow degradation of rhBChE-sCy7 OFF in the accumulation area (fluorescence increases to attain its maximum while zero activity of rhBChE in blood is detected).

The results of an *in vivo *biodistribution of rhBChEsCy7 ON and rhBChE-sCy7 OFF specimens in mouse organs are shown in *Fig. 4*. Both specimens mainly accumulate in the liver, kidneys, and bladder. The liver is the main organ responsible for degradation of the enzyme; maximum enhancement of integral fluorescence indicating enzyme biodegradation is observed 6 h after intravenous injection. The degradation products enter the blood flow and are eliminated mostly by the kidneys. Hence, *in vivo *distribution data of a proteinbased pharmaceutical mainly confirm the findings obtained at the previous stages (see *Fig. 2, 3 *and *Table 2*).

## CONCLUSIONS

Fluorescent probes are a simple and very sensitive method to detect proteolytic activity. The advances in the chemistry of fluorescent dyes have made it possible to design substrates with a high fluorescence intensity and low background signal. We designed a panel of fluorescent substrates based on proteins excessively labeled with sulfo-Cyanine5 and sulfo-Cyanine7 fluorophores and attained a 700-fold enhancement of fluorescence after proteolysis. The application of an excessively labeled specimen for *in vivo *imaging of the organs and tissues responsible for degradation and elimination of pharmaceuticals enable one to characterize the behavior of therapeutic protein-based pharmaceuticals in the organism more thoroughly. It is evident that researchers should take into account the pattern and site of degradation of a potential pharmaceutical when attempting to improve its pharmacokinetic parameters. The methods increasing blood retention together with the liver accumulation rate reduction may contribute to the design of pharmaceuticals with prolonged half-life. It should be mentioned that this approach is universal allowing one to perform *in vivo *degradation studies of any protein or peptide. However, excessive labeling may drastically change the pharmacokinetic properties and the elimination route of a certain protein-based pharmaceutical [24], which should be taken into consideration when choosing a fluorescent bioimaging technique during pharmacokinetic studies. The proposed approach may prove to be extremely topical during comparative preclinical studies using a panel of similar protein-based pharmaceuticals and selecting the optimal candidate that would exhibit the targeted effect.

## Abbreviations

SPECT: single-photon emission computed tomography
PET: positron emission tomography
HIV: human immunodeficiency virus
rhBChE: tetrameric recombinant human butyrylcholinesterase
PRAD: proline-rich attachment domain
MMP: matrix metalloproteinase
sCy5: Sulfo-Cyanine5
sCy7: Sulfo-Cyanine7
Fmax: relative fluorescence intensity enhancement
Fenz: fluorescence intensity enhancement after proteolytic digestion
F0: fluorescence intensity before proteolytic digestion
N: modification degree of pharmaceuticals
rhBChE-sCy7 ON: fluorescent rhBChE-sCy7 conjugate having no self-quenching effect
rhBChE-sCy7 OFF: nonfluorescent rhBChE-sCy7 conjugate having a quenching effect
BSA: bovine serum albumin
KLH: hemocyanin
